# Escalated heatwave mortality risk in sub-Saharan Africa under recent warming trend

**DOI:** 10.1126/sciadv.ady7379

**Published:** 2025-11-26

**Authors:** Cheng He, Yixiang Zhu, Yichen Guo, Jovine Bachwenkizi, Renjie Chen, Haidong Kan, Wafaie W. Fawzi

**Affiliations:** ^1^ Department of Global Health and Population, Harvard T.H. Chan School of Public Health, Boston, MA, USA.; ^2^ School of Public Health, Key Lab of Public Health Safety of the Ministry of Education, NHC Key Lab of Health Technology Assessment, IRDR ICoE on Risk. Interconnectivity and Governance on Weather/Climate Extremes Impact and Public Health, Fudan University, Shanghai, China.; ^3^ School of Nursing and Public Health, University of KwaZulu-Natal, Durban, South Africa.; ^4^ Department of Environmental and Occupational Health, Muhimbili University of Health and Allied Sciences, Dar es Salaam, Tanzania.; ^5^ Children’s Hospital of Fudan University, National Center for Children’s Health, Shanghai, China.; ^6^ Department of Epidemiology, Harvard T.H. Chan School of Public Health, Boston, MA, USA.; ^7^ Department of Nutrition, Harvard T.H. Chan School of Public Health, Boston, MA, USA.

## Abstract

Evidence from high-income countries indicates populations are adapting to frequent heatwaves, but similar trends in resource-constrained regions remain unknown. We analyzed mortality data from 11 Health and Demographic Surveillance Systems across sub-Saharan Africa (2005 to 2015) to examine temporal changes in heat-related mortality risk. Contrary to global trends, our findings suggest that heat vulnerability is increasing across African populations. Nighttime heatwave mortality risk increased significantly between 2005 to 2010 and 2011 to 2015 [OR from 1.02 (95% CI: 0.87 to 1.13) to 1.18 (95% CI: 1.13 to 1.23)], while daytime heatwaves showed no significant impact. Compound heatwaves transformed from nonsignificant to significant risk factors [OR = 1.11 (95% CI: 1.03 to 1.22)]. Males showed increased risks across all heatwave types, females only for nighttime and compound heat. Children under 5 showed universal risk increases, while the elderly showed the highest increases for nighttime and compound heat. These findings suggest that physiological adaptation alone is insufficient to cope with increasingly frequent heatwaves without adequate socioeconomic resources. Heightened nighttime vulnerability underscores the need for context-specific adaptations reflecting Africa’s distinct conditions.

## Teaser

Nighttime heat exposure is driving increased vulnerability to heatwaves across African populations amid recent warming.

## INTRODUCTION

Global average temperature has risen rapidly in recent years, making the past decade the hottest since pre-industrial times (*1*). This warming trend has driven a marked rise in the frequency, intensity, and duration of heatwaves (*2*). Africa has experienced some of the most pronounced warming trends globally, with temperatures increasing at approximately 1.5 times the global rate (*3*, *4*). The health impacts of heatwaves are significant, with direct effects on heat stroke (*5*), heat exhaustion, and dehydration (*6*), while also affecting underlying cardiovascular (*7*) and respiratory diseases (*8*). Although heat-related mortality is likely to be severe in resource-limited settings due to occupational heat exposure (*9*), limited cooling infrastructure, and deficiency healthcare systems (*10*, *11*), few studies have comprehensively assessed heat-related health outcomes across sub-Saharan Africa.

The concept of population adaptation to heat exposure has gained attention recently, with evidence from countries in North America, East Asia, and Europe suggesting decreasing heat-related health risks over time as extreme heatwave events become increasingly frequent, a phenomenon attributed to physiological acclimatization, behavioral changes, improved housing, and strengthened public health systems (*12*–*14*). However, the applicability of this adaptation changes to African populations remains questionable, as the socioeconomic contexts differ substantially, with limited access to cooling technologies (*15*), unreliable electricity supply (*16*), and inadequate housing infrastructure (*17*). Furthermore, while studies from high-income regions suggest greater heat vulnerability among women and the elderly (*12*), these patterns may not translate to African settings with different gender-based occupational roles and housing conditions. Men in sub-Saharan Africa often engage in physically demanding outdoor labor with high heat exposure, while women’s domestic responsibilities may involve prolonged exposure to poorly ventilated environments (*18*). In addition, emerging evidence indicates that the timing of heat exposure, day or night, may lead to different physiological effects (*19*). Heat exposure during the day might mainly affect outdoor activity, while nighttime heat might compromise the body’s recovery processes during sleep (*20*). While the evidence is growing on whether nighttime temperatures are increasing faster in many parts of Africa (*21*), few studies have differentiated the health impacts of day or night heat exposure to date.

In all, the knowledge gaps regarding differential heat vulnerability across population subgroups are compounded by methodological limitations in existing research, including the predominant focus on daytime temperature metrics, limited temporal analyses of adaptation patterns, and insufficient demographic stratification. These research gaps impede the development of targeted heat-health interventions across the continent, where climate change impacts are expected to be severe but adaptive capacity remains constrained.

To address these critical gaps here, we aim to first assess the differential mortality impacts of daytime, nighttime, and compound (day-night) heatwaves across diverse African populations. Second, we plan to evaluate temporal changes in heat-related mortality risk by comparing two distinct periods in order to test the heat adaptation changes in the African context during a period of accelerating warming. Third, we plan to identify demographic variations in heat vulnerability by conducting stratified analyses across gender and age groups, therefore providing insights into population-specific risk patterns. By integrating mortality data from 11 demographic surveillance sites across eight countries with high-resolution temperature data, our analysis provides insights into heat-health relationships across sub-Saharan Africa, with implications for climate adaptation planning and public health policy in the region.

## RESULTS

**Fig. 1.**
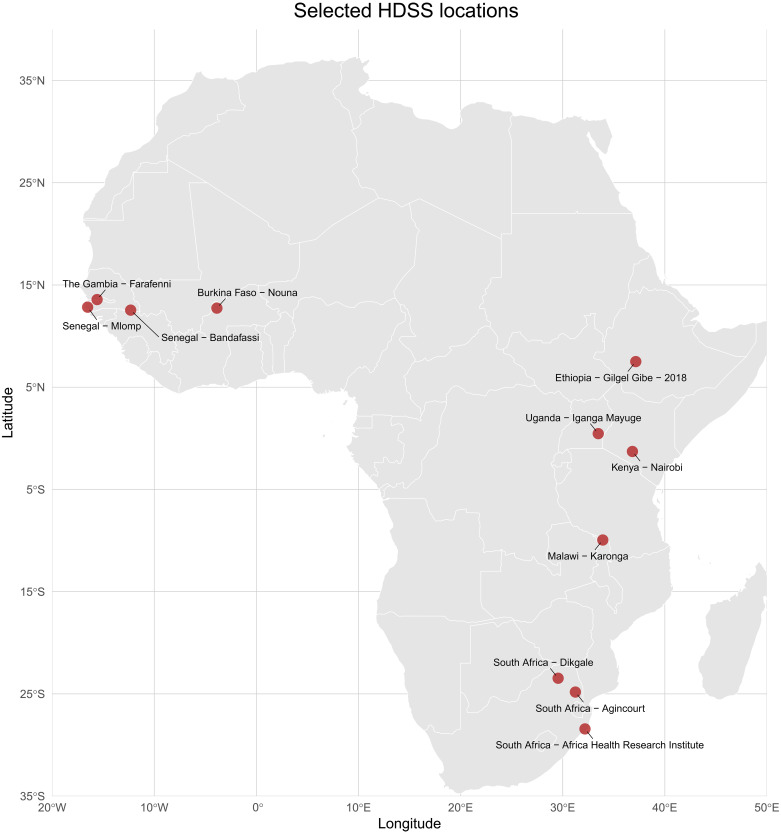
Geographical distribution of HDSS surveillance sites included in the study.

The study examined the mortality data from 11 Health and Demographic Surveillance System (HDSS) sites across eight countries in sub-Saharan Africa (Fig. 1). The surveillance sites had a wide range of geographic locations, with varied climate zones in West Africa (Senegal-Bandafassi, Senegal-Mlomp, The Gambia–Farafenni, and Burkina Faso–Nouna), East Africa (Ethiopia–Gilgel Gibe, Uganda–Iganga Mayuge, Kenya-Nairobi, and Malawi-Karonga), and Southern Africa (South Africa–Dikgale, South Africa–Agincourt, and South Africa–Africa Health Research Institute). This broad geographic representation includes a wide variety of climate regimes and socioeconomic settings across sub-Saharan Africa. The total dataset of mortality cases consisted of 50,289 cases that occurred from 2005–2015 with a similar distribution for the two periods of analysis: 23,542 deaths (46.8%) during the period of 2005–2010, and 26,747 deaths (53.2%) from 2011–2015 (Table 1). The demographic characteristics of all cases were also similar in both analysis periods (table S1). Age distribution showed working-age adults (18 to 65 years) comprised the highest proportion of mortality cases (44.2%) followed by elderly (>65 years, 23.8%), children aged 5 to 18 years (18.0%), and young children (≤5 years, 14.0%).

**Table 1.** Demographic characteristics of mortality cases across study periods, 2005 to 2015. Percentages indicate the proportion of cases within each time period. Age groups are defined as: young children (≤5 years), school-age children (5 to 18 years), working-age adults (18 to 65 years), and elderly (>65 years). Data were collected from 11 HDSS surveillance sites across eight countries in sub-Saharan Africa.

	Total	2005–2010	2011–2015
**All death**	50,289	23,542	26,747
**Sex**			
Male	25,816 (51.3%)	12,035 (51.1%)	13,781 (51.5%)
Female	24,473 (48.7%)	11,508 (48.9%)	12,965 (48.5%)
**Age**			
≤5	7,028 (14.0%)	3,454 (14.7%)	3,574 (13.4%)
5–18	9,074 (18.0%)	4,432 (18.8%)	4,642 (17.4%)
18–65	22,204 (44.2%)	10,784 (45.8%)	11,420 (42.7%)
>65	11,983 (23.8%)	4,872 (20.7%)	7,111 (26.6%)

**Fig. 2.**
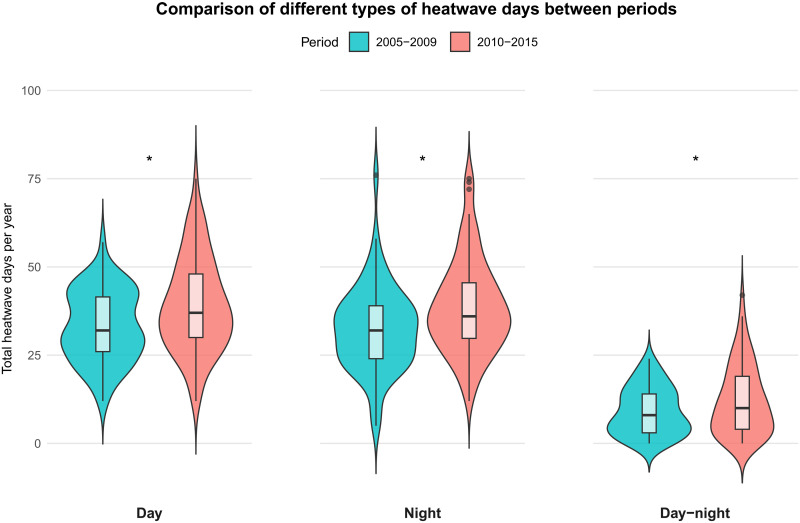
Comparison of heatwave days by type between time periods. Violin plots showing the distribution of annual heatwave days across study sites for three heatwave types (daytime, nighttime, and day-night compound) comparing 2005 to 2009 (teal) and 2010 to 2015 (coral) periods. Box plots within each violin show the median (horizontal line), IQR (box), and outliers. All three heatwave types show significant increases in the later period, with asterisks (*) indicating significant differences between periods (*P* < 0.05).

Analysis of heatwave occurrence patterns showed significant changes in exposure frequencies for all three heatwave types (Fig. 2 and table S2). There were consistent increases in the annual frequency of all three heatwave categories between the early (2005 to 2009) and later (2010 to 2015) periods. The median number of daytime heatwave days per year increased from 32 [interquartile range (IQR): 26 to 41.5] to 37 (IQR: 30 to 48) (*P* = 0.01375), nighttime heatwave frequency showed a significant increase from 32 (IQR: 24 to 39) to 36 (IQR: 30 to 46) days per year (*P* = 0.00755), and compound heatwave events demonstrated a statistically significant increase from 8 (IQR: 3 to 14) to 10 (IQR: 5 to 19) days per year (*P* = 0.02567). These data suggest a systematic increase in extreme heat exposure in sub-Saharan Africa during the period of study.

**Fig. 3.**
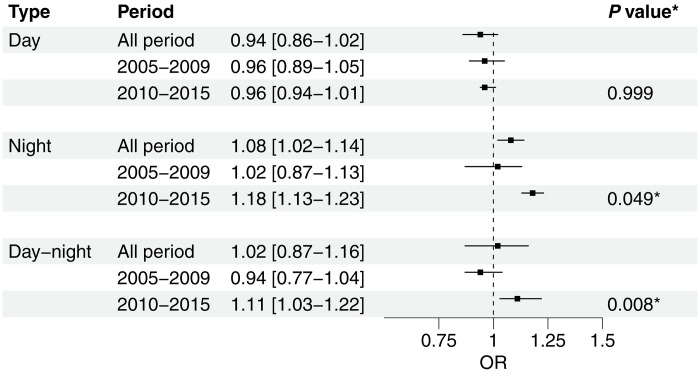
Heat-related mortality risk by time period for different heatwave types. Plot showing odds ratios (OR) with 95% confidence intervals (CIs) for mortality risk associated with three heatwave types (daytime, nighttime, and day-night compound) across different time periods. Results show cumulative effects over lag 0 to 6 days, after controlling for daily mean temperature effects. *P* values indicate significance of temporal change between periods. The dashed vertical line at OR = 1 represents the null hypothesis of no effect, with asterisks (*) indicating significant differences between periods (*P* < 0.05).

The analysis of differentials in mortality risk across heatwave types demonstrated notable temporal variations in vulnerability trends (Fig. 3). Daytime-only heatwaves showed no significant association with mortality risk during either time period {2005 to 2009: odds ratio (OR) = 0.96 [95% confidence interval (CI): 0.89 to 1.05]; 2010 to 2015: OR = 0.96 (95% CI: 0.94 to 1.01)}. In contrast, nighttime heatwaves demonstrated a significant temporal shift in mortality risk, with minimal impact during 2005 to 2009 [OR = 1.02 (95% CI: 0.87 to 1.13)] but substantially increased risk during 2010 to 2015 [OR = 1.18 (95% CI: 1.13 to 1.23)], representing a statistically significant change between periods (*P* = 0.049). Similarly, compound day-night heatwaves showed a significant escalation in mortality impact, transitioning from a nonsignificant protective association in 2005 to 2009 [OR = 0.94 (95% CI: 0.77 to 1.04)] to a significant risk factor in 2010 to 2015 [OR = 1.11 (95% CI: 1.03 to 1.22)], with this temporal change being highly significant (*P* = 0.008). Lag-response patterns (figs. S1 to S3) further confirmed this distinctive temporal shift, with nighttime and compound heatwaves showing immediate acute effects (0- to 1-day lag) in the 2010 to 2015 period that were absent in the earlier period. Region analyses for West, East, and South Africa (table S3) showed generally consistent increases in mortality risk in the later period. Although some estimates did not reach statistical significance because of smaller case numbers within individual regions, the overall temporal patterns were consistent, indicating that the observed changes were not driven by any single region.

**Fig. 4.**
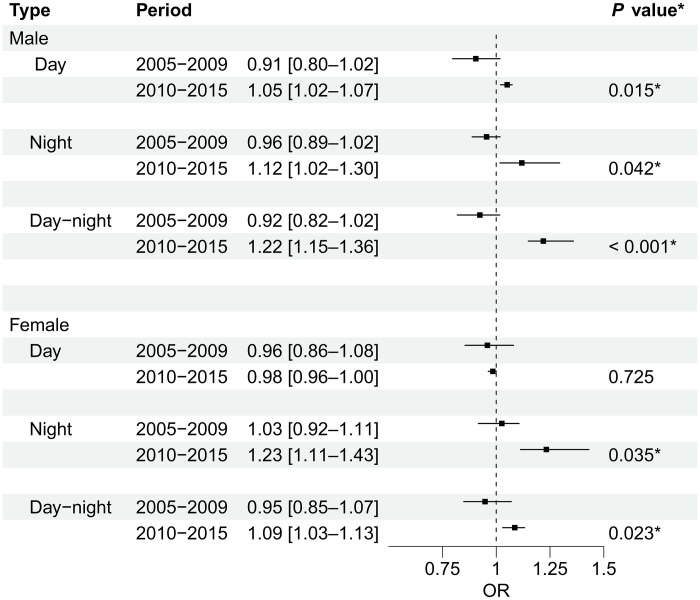
Gender-stratified heat-related mortality risk by time period and heatwave type. Plot showing odds ratios (OR) with 95% confidence intervals (CIs) for mortality risk associated with three heatwave types (daytime, nighttime, and day-night compound) across different time periods. Results show cumulative effects over lag 0 to 6 days, after controlling for daily mean temperature effects. P-values indicate significance of temporal change between periods. The dashed vertical line at OR = 1 represents the null hypothesis of no effect, with asterisks (*) indicating significant differences between periods (*P* < 0.05).

Gender-stratified analyses revealed distinct patterns of heat vulnerability between males and females (Fig. 4). Males exhibited significant increases in mortality risk across all heatwave types between the two time periods. For daytime heatwaves, male vulnerability transitioned from a nonsignificant protective effect in 2005 to 2009 [OR = 0.91 (95% CI: 0.80 to 1.02)] to a significant risk in 2010 to 2015 [OR = 1.05 (95% CI: 1.02 to 1.07); *P* = 0.015]. Male vulnerability to nighttime heatwaves similarly increased from a nonsignificant association [OR = 0.96 (95% CI: 0.89 to 1.02)] to a significantly elevated risk [OR = 1.12 (95% CI: 1.02 to 1.30); *P* = 0.042]. Most notably, males showed the strongest temporal increase in vulnerability to compound day-night heatwaves, with odds ratios rising from 0.92 (95% CI: 0.82 to 1.02) to 1.22 (95% CI: 1.15 to 1.36) (*P* < 0.0001). In contrast, females showed a more selective vulnerability pattern, with no significant change in daytime heatwave risk between periods, while demonstrating significant increases in vulnerability to nighttime heatwaves [OR = 1.03 (95% CI: 0.92 to 1.11) to OR = 1.23 (95% CI: 1.11 to 1.43); *P* = 0.035] and compound heatwaves [OR = 0.95 (95% CI: 0.85 to 1.07) to OR = 1.09 (95% CI: 1.03 to 1.13); *P* = 0.023]. As shown in table S4, during the 2010 to 2015 period, the impact of daytime heatwaves and compound heatwaves on males was significantly higher than on females (*P* < 0.05).

**Fig. 5.**
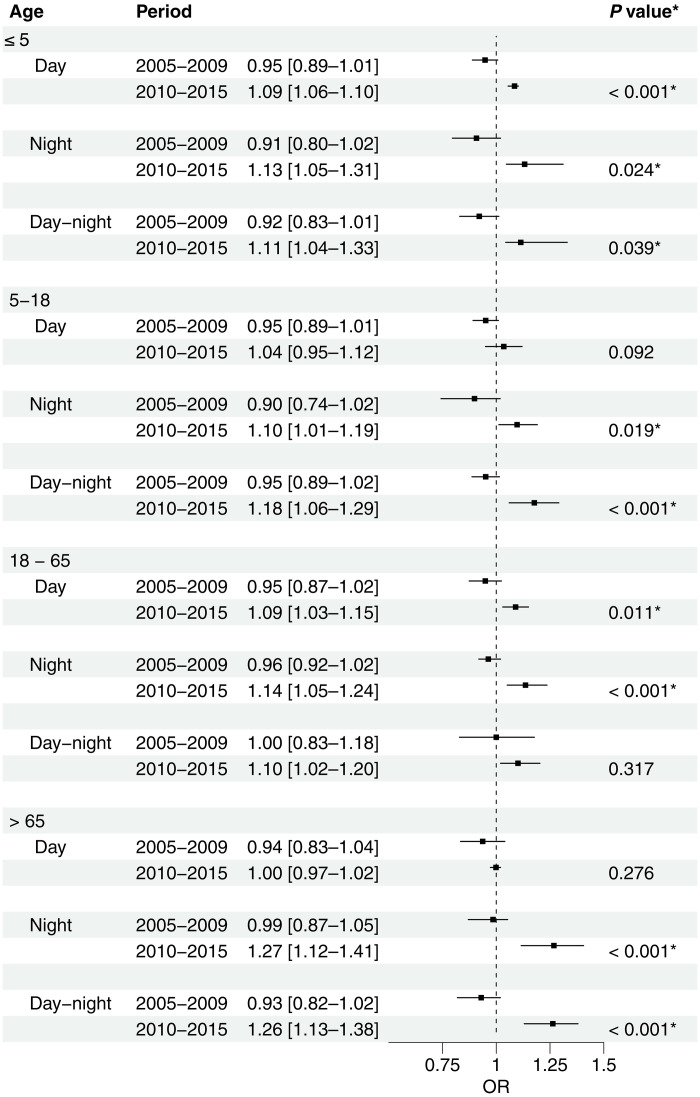
Age-stratified heat-related mortality risk by time period and heatwave type. Plot showing odds ratios (OR) with 95% confidence intervals (CIs) for mortality risk associated with three heatwave types (daytime, nighttime, and day-night compound) across different time periods. Results show cumulative effects over lag 0 to 6 days, after controlling for daily mean temperature effects. *P* values indicate significance of temporal change between periods. The dashed vertical line at OR = 1 represents the null hypothesis of no effect, with asterisks (*) indicating significant differences between periods (*P* < 0.05).

Age-stratified analyses revealed distinct vulnerability patterns across different life stages (Fig. 5). Young children (≤5 years) demonstrated significantly increased vulnerability to all heatwave types between the two time periods. School-age children (5 to 18 years) showed significant increases in vulnerability primarily to nighttime heatwaves [OR = 0.90 (95% CI: 0.74 to 1.02) to OR = 1.10 (95% CI: 1.01 to 1.19); *P* = 0.019] and compound heatwaves [OR = 0.95 (95% CI: 0.89 to 1.02) to OR = 1.18 (95% CI: 1.06 to 1.29); *P* < 0.001]. Working-age adults (18 to 65 years) exhibited significant risk increases for daytime heatwaves [OR = 0.95 (95% CI: 0.87 to 1.02) to OR = 1.09 (95% CI: 1.03 to 1.15); *P* = 0.011] and nighttime heatwaves [OR = 0.96 (95% CI: 0.92 to 1.02) to OR = 1.14 (95% CI: 1.05 to 1.24); *P* < 0.001]. Most notably, the elderly population (>65 years) showed the most pronounced vulnerability increases for nighttime heatwaves [OR = 0.99 (95% CI: 0.87 to 1.05) to OR = 1.27 (95% CI: 1.12 to 1.41); *P* < 0.001] and compound heatwaves [OR = 0.93 (95% CI: 0.82 to 1.02) to OR = 1.26 (95% CI: 1.13 to 1.38); *P* < 0.001], while showing no significant change in vulnerability to daytime-only heat exposure. As shown in table S5, during the 2010 to 2015 period, the impact of nighttime heatwaves and compound heatwaves on the elderly population (>65 years) was significantly higher than other age group (*P* < 0.05).

Our findings demonstrated robustness across multiple sensitivity analyses (table S7). Adjusting for PM_2.5_ produced minimal changes in effect estimates, with nighttime and compound heatwave associations remaining significant in 2010 to 2015. Alternative model specifications (df = 5 for lag response) yielded consistent results. When applying a more stringent heatwave definition (95th percentile threshold), as shown in table S7, we observed stronger effect estimates for all heatwave types in the later period, further confirming the temporal increase in heat vulnerability.

## DISCUSSION

This study reports evidence that heat-related mortality risks have significantly changed across sub-Saharan Africa over the past decade, which has important implications for climate and health adaptation strategies. We found that, after controlling for the effect of daily mean temperature, while daytime-only heatwaves demonstrated weak effects across both time periods, nighttime heatwaves and compound heatwaves exhibited significant increases in mortality risk from 2005–2009 to 2010–2015. So, the increases in vulnerability over time were stronger for nighttime heatwaves and compound heatwaves (in contrast to daytime heatwaves). These findings contrast with the adaptation change seen previously in high-income regions, indicating that populations in Africa could be experiencing increased vulnerability to extreme heat events, as global warming continues. The differentiated effects by demographic group, males experiencing increasing vulnerability to all types of heatwaves, working-age adults with increasing risk for both daytime and nighttime heatwaves, and the elderly experiencing higher increases in risk from nighttime heatwaves, may reflect the complex interplay of physiological, behavioral, and socioeconomic factors that shape heat vulnerability in sub-Saharan Africa.

Compared to existing studies, overall, our findings contrast with heat vulnerability trends observed in developed regions, where multiple studies have documented decreasing risk over time. The study in the United States showed a significant reduction in heat risk from the period of 1973–1982 to 2003–2013 (*12*), while similar adaptation trends have been observed in Europe (*13*) and East Asia (*22*), attributed to increased air conditioning prevalence, improved healthcare access, and enhanced public awareness. However, our observation of increasing heat vulnerability aligns with limited evidence from other developing regions, including recent studies from India and Southeast Asia that suggest increasing heat-related risks (*23*). In particular, our findings regarding the heightened impact of nighttime heat exposure extend previous physiological research demonstrating that nocturnal temperatures critically affect human thermoregulation and sleep quality (*24*). While studies have examined daytime heat effects in African settings (*25*), our work represents one of the first to systematically differentiate between daytime, nighttime, and compound heat exposures across diverse African populations. In addition, our gender-stratified findings challenge traditional assumptions about female heat vulnerability predominance, commonly cited in European and North American literature (*5*, *12*), and instead reveal substantial male vulnerability, particularly to daytime heat exposure, which may reflect region-specific occupational and behavioral patterns. This comprehensive demographic and exposure-time analysis provide critical evidence that heat adaptation trends observed in developed economies cannot be assumed universal, with significant implications for climate change health impact projections in Africa. Compared with previous studies that have assessed temporal changes in population adaptation over several decades (*12*, *22*), our analysis covers a shorter 10-year period. A longer study duration would provide more robust evidence of adaptation trends, but due to data availability across multiple sites, this was the maximum period available. Nevertheless, the rapid rate of warming (*26*), together with the significant increases we observed in heatwave frequency, and the limited adaptive capacity in many communities in sub-Saharan Africa suggest that meaningful shifts in heat-related vulnerability may still emerge within this timeframe.

The identified differential impact of heatwaves, specifically the more pronounced impact of nighttime heat, can be attributed to various physiological and environmental pathways. Nighttime temperatures are critical for thermoregulation as they allow for the thermal recovery from daytime heat stress, and when nighttime temperatures remain elevated, the reduced capability of the body to lower core temperatures may have a secondary impact on sleep duration and increase cardiovascular strain (*5*, *24*). This lack of thermal recovery may have an even greater impact in Africa, where there are fewer instances of air conditioning, where approximately 110 million rural residents lack access to electricity, and 630 million urban residents do not have access to reliable or affordable electricity (*27*). In other words, despite witnessing increases in heatwave frequency, a majority of heat-exposed populations are unable to access air conditioning or even basic cooling devices such as fans. When residents are exposed to compound heat events, their reliance on passive cooling measures becomes ineffective. The increase in vulnerability from 2005–2009 to 2010–2015 may be indicative of a threshold effect, whereby increasing minimum temperatures have resulted in exceeding tolerance thresholds more frequently. Repeat exposures to ambient heat without an adequate period for thermal recovery create cumulative physiological stress such as dehydration, electrolyte imbalance, and increased cardiovascular strain that can ultimately lead to death (*19*), especially for those with existing health issues. Furthermore, residents of impoverished backgrounds unable to purchase air conditioning spend more time outdoors attempting to cool during heatwave events, increasing their exposure to disease pathogens, and the compromised living conditions are typically characterized as overcrowding and poor ventilation (*28*). These interactions show how heat exposure becomes a significant risk factor through multiple complex physiological and environmental pathways and potentially increases as recent warming progresses.

The demographic patterns in our findings reveal important insights about heat vulnerability in the African context. Gender-specific vulnerability patterns likely reflect a combination of occupational exposures and social factors. On the one hand, males in many African settings are engaged in outdoor labor (agriculture, construction, and mining) that increases daytime heat exposure (*29*), which may underlie their significant vulnerability increase across all heatwave types. On the basis of the 2020 ILOSTAT data from International Labour Organization, approximately 60% of male workers in East Africa are employed in agriculture, compared to 40% of females (*30*), and men generally carry out physically demanding fieldwork tasks such as plowing during peak daylight hours (*31*). The Quarterly Labour Force Survey from South Africa indicated that 88% of construction workers and 85% of miners are male, working during daytime, and are therefore exposed to extreme heat while working in direct sunlight (*32*). Studies confirmed that male workers in agriculture and mining are at particular risk during extreme heat events due to extended exposure and limited access to cooling (*28*). Conversely, while women appeared to be at less risk from daytime heat, they exhibited considerable risk increases from nighttime heat exposure due to their likely role in indoor domestic activities, where heat exposure is prolonged in substandard housing conditions (*33*). Age-stratified patterns found that older populations demonstrated especially raised risks from nighttime heat exposure, which aligns with declines in thermoregulatory capacity, nocturnal sweating ability, and cardiovascular efficiency, that occur with aging (*34*). Vulnerability to nighttime heat across all exposure types increased among children under the age of 5 years, possibly reflecting their dependency on caregivers to initiate heat adaptation behaviors as well as their continuing thermal regulation development (*25*).

Our findings indicate the need for urgent and context-specific policy interventions for the African context. Current heat early warning systems in Africa, particularly in the West African region, rely on indicators such as the heat index (average temperature and humidity) and current-day maximum temperature, while none of them consider nighttime temperature (*35*). Meteorological services in countries like Ghana and Nigeria typically issue heat warnings based on maximum temperatures during the daytime and thus do not consider the important health impacts of rising nighttime temperatures (*36*). More concerning is the World Health Organization’s finding that approximately 60% of Africa’s over 1.2 billion population remains unprotected by any form of early warning systems (*37*). Therefore, our research highlights the important role of early warning systems in a warming world, as mentioned before, which should specifically include nighttime temperature forecasts. Second, in terms of infrastructure development, development should focus on passive cooling approaches geared toward locations with little to no electricity access, including tree planting for shade (*38*), reflective cool roofing materials (*39*), and improved housing designs that can lessen nighttime heat stress. Third, community adaptation programs should be designed to reach the identified vulnerable populations, especially the elderly and young children, with cooling centers made available during heat events. Fourth, safety at work regulations should be more stringent when protecting outdoor workers, particularly males that were vulnerable to all three types of heatwaves. Ultimately, our findings underscore that adaptation strategies should not rely on assumptions based on high-income country experiences, it should instead incorporate the facts on the ground that reflect African socioeconomic realities. With limited air conditioning access, unreliable electricity, and inadequate housing insulation, technological solutions common elsewhere may not be viable. International climate financing groups should recognize this adaptation gap and prioritize context-appropriate heat-health interventions as a matter of climate justice, particularly as African regions have contributed minimally to global greenhouse gas emissions while facing disproportionate impacts.

Several limitations of this study should also be mentioned. First, while HDSS surveillance sites provide valuable mortality data, they may not fully represent the demographic and environmental heterogeneity across the whole African continent. Second, though widely validated, ERA5-Land reanalysis data may not capture local microclimatic conditions that influence individual heat exposure, especially indoor temperature conditions, which may introduce bias. Third, our analysis lacks more critical individual socioeconomic variables, so could not fully account for important socioeconomic factors and adaptive behaviors that likely modify heat vulnerability, which could provide better guidance for future intervention studies. These limitations highlight the need for expanded surveillance systems with enhanced environmental and socioeconomic data collection to better understand heat-health relationships in African contexts.

In conclusion, our findings suggest that heat-related mortality risk has increased across the sub-Saharan African region recently, particularly for nighttime and compound heatwaves. The temporal change in vulnerability patterns reflects contrasting trends for adaptation in high-income regions and highlights critical differences in vulnerability across demographic groups. Males exhibited increased vulnerability across all heatwave types, and the elderly demonstrated the most pronounced risk increasing specifically for nighttime heat exposure, and children showed universal vulnerability increases. These disproportionate impacts from nighttime heat suggest that increasing minimum temperatures may be an underestimated risk to public health in African contexts. Overall, these results call for an urgent need for context-specific adaptation strategies that consider the specific socioeconomic, infrastructural, and environmental conditions of sub-Saharan Africa.

## MATERIALS AND METHODS

### Mortality datasets

We obtained mortality data from the INDEPTH HDSS network (*40*). The INDEPTH HDSS mortality data collection protocol involves systematic monthly household visits, during which trained fieldworkers document deaths and gather cause-of-death information through standardized verbal autopsy procedures (*41*). Data are gathered on the basis of structured interviews of the deceased’s relatives to record symptoms related to death. The specific causal attribution is conducted by either a physician review process or by using a validated algorithmic methodology. The data are subjected to rigorous validation on multiple levels of quality control, including random field re-interviews, verification of logical consistency, duplicate detection protocols, and comprehensive missing value assessments (*41*). Additional validation includes cross-referencing any available medical records, official statistics, and community reports (*41*). These datasets have been extensively validated and widely used in epidemiological research (*42*–*44*).

Our analysis initially incorporated all 37 potential datasets from multiple HDSS sites across sub-Saharan Africa. From this complete HDSS dataset, we selected sites that offered the longest overlapping temporal coverage of mortality records, resulting in a final dataset comprising 11 surveillance sites across 8 countries for the period from 2005 to 2015. For each HDSS location, we obtained precise geographical coordinates. Individual mortality records included exact age at death, calculated from documented birth and death dates in the HDSS datasets, alongside demographic information including sex.

On the basis of our previous research framework (*5*), and to analyze the temporal change of recent global warming effects, we separated our analysis into two time periods: 2005 to 2010 and 2011 to 2015. This structure allowed us to systematically assess for potential changes in the vulnerability of the population to heat exposure over time.

### Heatwave exposure assessment

Considering the limited meteorological station data available close to the selected surveillance sites, we used the ERA5-Land reanalysis dataset to represent ambient temperature exposure. This high-resolution gridded dataset, produced by the European Centre for Medium-Range Weather Forecasts, offers hourly temperature outputs at 0·1 by 0·1° spatial resolution (*45*). The ERA5-Land dataset uses advanced data assimilation methods to generate temperature outputs that are closely aligned with station observations (*45*). The validity of ERA5-Land data for epidemiological studies has been widely documented in multiple comparative studies (*46*, *47*). To further ensure accuracy for our study sites, we additionally validated ERA5-Land estimates against station observations located within 50 km of selected HDSS sites, obtained from the Global Historical Climatology Network. Because only three stations (Nairobi-Wilson, Senegal-Kedougou, and South Africa–Skukuza) had complete records during 2005 to 2015 within this distance, these sites were included in the validation. As presented in table S8, ERA5-Land temperatures showed high correlations (*r* = 0.79 to 0.92) and acceptable errors (root mean square error mostly 1° to 2°C) compared with ground measurements, supporting their suitability for use in our analysis. Given this, we extracted hourly air temperature series for all selected HDSS locations using the closet grid cells from the ERA5-Land dataset to represent local temperature exposure conditions. After adjusting for different time zones for each of the surveillance sites, we calculated daily mean, maximum, and minimum temperatures for each study location during the period of 2005 to 2015.

To identify the distinctive impacts of heatwaves occurring during different diurnal periods and extend our recent work (*19*), we implemented three separate heatwave classifications. Daytime heatwaves were defined as days when maximum daily temperatures exceeded the local 90th percentile threshold for two or more consecutive days. Nighttime heatwaves were categorized as periods when minimum daily temperatures surpassed the local 90th percentile threshold for at least two consecutive days. Day-night heatwaves were designated as events meeting both daytime and nighttime criteria concurrently. We performed site-specific heatwave identification procedures for each surveillance location based on local temperature distributions observed throughout the 2005 to 2015 study period.

### Statistical analysis

The mortality impact of heatwave exposure was investigated using a time-stratified case-crossover study design (*48*, *49*). Within this methodological framework, we defined the date of death as the case day for each mortality event. Control days were selected from the same month and year as the case day while matching the specific day of the week. This approach enables each case to serve as its own control, effectively accounting for individual-level covariates (including sex, age, and socioeconomic factors) that remain stable over short time periods. The time-stratified case-crossover design offers particular advantages for environmental epidemiology, as it inherently controls for potentially confounding influences arising from weekly patterns, seasonal fluctuations, and long-term temporal trends (*49*, *50*). By matching case and control days within relatively narrow time windows, we minimized the risk of time-varying confounders while maintaining statistical efficiency in estimating acute heat exposure effects on mortality outcomes across diverse African settings.

We implemented a conditional logistic regression as our main analytical model. In this model, mortality events were treated as the dependent variable, with heatwave occurrence (represented as a binary indicator) serving as the independent variable. To account for the delayed effects of heatwave exposure, we incorporated a distributed lag model (*51*). On the basis of our previous research (*5*) and the natural setting of the case-crossover design, we selected a maximum lag period of 7 days (0 to 6 days). The lag-response relationship was modeled using a natural cubic B-spline with three degrees of freedom (df), with internal knots positioned at equally spaced values on the lag scale (*52*, *53*). In this main model, we additionally controlled for daily mean temperature effects using a distributed lag nonlinear model (DLNM) (*54*). Specifically, we constructed a cross-basis function for daily mean temperature incorporating a quadratic B-spline with default equally spaced knots, consistent with methodological approaches in previous studies (*5*, *53*). The lag response of this cross-basis function was modeled using a natural cubic B-spline with four degrees of freedom and internal knots positioned at equally spaced values on the lag scale, maintaining the same maximum lag duration of 7 days (*53*).

To assess potential temporal changes in heatwave impacts, we conducted separate analyses for all three heatwave types across two distinct periods: 2005 to 2009 and 2010 to 2015. To evaluate whether differences between these two time periods were statistically significant, we implemented the multivariate Wald test, with *P* values below 0.05 considered statistically significant (*5*, *55*). For comprehensive methodological details regarding this statistical comparison approach, please refer to the Supplementary Materials. Furthermore, we performed stratified analyses based on age categories and gender (male and female) to investigate potential demographic variations in heat vulnerability.

To test the robustness of our findings, we conducted several sensitivity analyses. First, we considered an alternative definition of the heatwave by varying intensity thresholds (using the 95th percentile). Second, we added natural splines for air pollutants, primarily PM_2.5_, to the main model to control for the potential confounding that may result from air pollution. Third, we systematically checked the key model parameters to ensure the stability of our results under different analytic conditions. Details of these sensitivity analyses are described in the Supplementary Materials.

All these statistical analyses were completed using R software. The results represent odds ratios with 95% CI to estimate the relative risk of mortality during heatwave days compared with non-heatwave days.

## Supplementary Materials

### This PDF file includes:

Methods for testing the statistical significance of the difference Tables S1 to S8 Figs. S1 to S3 References
