# Supplementary material for: Escalated heatwave mortality risk in sub-Saharan Africa under recent warming trend
Source: Sci Adv. 2025 Nov 26;11(48):eady7379. doi: 10.1126/sciadv.ady7379 (PMC12652248; doi:10.1126/sciadv.ady7379)
Supplement: Supplementary file 1 — Methods for testing the statistical significance of the difference Tables S1 to S8 Figs. S1 to S3 References [file sciadv.ady7379_sm.pdf]

Science Advances
AAAS

Supplementary Materials for

Escalated heatwave mortality risk in sub-Saharan Africa under recent warming trend

Cheng He *et al.*

Corresponding author: Cheng He, chenghe@hsph.harvard.edu

Sci. Adv. **11**, eady7379 (2025)
DOI: 10.1126/sciadv.ady7379

This PDF file includes:

Methods for testing the statistical significance of the difference
Tables S1 to S8
Figs. S1 to S3
References

Methods for testing the statistical significance of the difference

To test the statistical significance of the difference in odds ratios (ORs) between the two time periods over different subgroups, according to a related study (56), we calculated the z score as:

$$z = \frac{(E_1 - E_2)}{\sqrt{(SE_1)^2 + (SE_2)^2}}$$

Where E_1 and E_2 are the natural logarithms of the estimated OR, SE_1 and SE_2 are their respective standard errors calculated from their estimated 95% CIs.

Sensitivity analysis

As documented in our methodology, we implemented multiple robustness checks to validate our findings through various aspects, encompassing alternative model specifications and diverse parameter selections.

(1) Definition of heat waves

In addition to the definition used in the main analysis (90th percentile 2 consecutive days), we used an alternative threshold (the 95th percentile) to define three different heat waves.

(2) Confounder effect of air pollution

To assess potential effect modification by air pollutants on different heatwave impacts, we incorporated key air pollutant measurements—primarily $PM_{2.5}$—into our main model as separate covariates. Daily mean $PM_{2.5}$ concentrations were extracted from the Long-term Gap-free High-resolution Air Pollutants (LGHAP) concentration dataset (57). This comprehensive database synthesizes multiple data sources including satellite aerosol optical depth measurements from various platforms (MODIS, VIIRS, MISR), MERRA-2 aerosol diagnostics, ERA5 reanalysis data, and ground-based measurements (57). The dataset employs advanced machine learning techniques, specifically tensor-flow-based gap-filling with attention mechanisms and SCene-Aware ensemble learning Graph ATtention network approaches, to generate global $PM_{2.5}$ concentration estimates. Validation against ground measurements demonstrates the dataset's exceptional accuracy, with a correlation coefficient of R=0.95 and root mean square error of 5.7μg/m^3.

(3) Key parameters in the main model

As suggested by the related temperature effect study (58), some settings of the key parameters in our main model may affect the estimate results, such as the knots setting, so, we adjusted the knots for exposure-response accordingly and to see if our findings were still stable.

Table S1 Distribution of Mortality Cases by Health and Demographic Surveillance System Sites Across Sub-Saharan African Countries, 2005-2015

Countries	Site names	2005-2010	2011-2015
Burkina Faso	Nouna	3,656	4,283
Ethiopia	Gilgel Gibe	1,657	2,282
Kenya	Nairobi	2,231	2,463
Malawi	Karonga	1,466	1,383
Senegal	Bandafassi	808	805
Senegal	Mlomp	317	382
South Africa	Africa Health Research Institute	4,436	3,671
South Africa	Agincourt	4,585	4,947
South Africa	Dikgale	384	1,724
The Gambia	Farafenni	1,667	1,906
Uganda	Iganga Mayuge	2,335	2,901
	**Total**	23,542	26,747

Table S2 Temporal Comparison of Annual Heatwave Frequency by Type Across Sub-Saharan African Study Sites, 2005-2015. P-values represent statistical significance of differences between time periods.

	**2005-2009**	**2010-2015**	***P*-value**
Number of daytime heatwave days per year	32 [26, 41.5]	37 [30, 48]	0.01375
Number of nighttime heatwave days per year	32 [24, 39]	36 [30, 46]	0.00755
Number of compound heatwave days per year	8 [3, 14]	10 [5, 19]	0.02567

Table S3 Regional Differences in Heatwave-Associated Mortality Risk Between Two Time Periods (2005-2009 vs 2010-2015) Data are presented as odds ratios with 95% confidence intervals in brackets. Results show the cumulative effects over lag 0-6 days for different heat wave types (daytime, nighttime, and compound) in 2005-2009 and 2010-2015. All odds ratios represent the comparison between heat wave days and non-heat wave days. *P-value* represents the statistical significance of the difference between two time periods.

	**Type**	**2005-2009**	**2010-2015**	***P*-value**
West Africa	Day Heatwave	0.94 [0.83, 1.08]	0.90 [0.80, 1.01]	0.493
	Night Heatwave	0.91 [0.81, 1.03]	1.15 [1.03, 1.29]	< 0.001**
	Day-Night Heatwave	0.82 [0.69, 1.02]	0.94 [0.81, 1.06]	0.110
East Africa	Day Heatwave	0.93 [0.81, 1.08]	1.03 [0.96, 1.11]	0.079
	Night Heatwave	1.05 [0.95, 1.15]	1.10 [1.00, 1.20]	0.329
	Day-Night Heatwave	0.85 [0.66, 1.08]	1.02 [0.86, 1.22]	0.094
South Africa	Day Heatwave	0.96 [0.82, 1.10]	1.06 [0.97, 1.15]	0.106
	Night Heatwave	0.97 [0.91, 1.01]	1.11 [1.02, 1.21]	< 0.001**
	Day-Night Heatwave	0.90 [0.78, 1.03]	1.24 [1.10, 1.40]	< 0.001**

Note: West Africa includes Burkina Faso-Nouna, Senegal-Mlomp, Senegal-Bandafassi, and The Gambia-Farafenni. East Africa includes Kenya-Nairobi, Uganda-Iganga Mayuge, and Ethiopia-Gilgel Gibe. South Africa includes South Africa-Dikgale, South Africa-Africa Health Research Institute, South Africa-Agincourt, and Malawi-Karonga. ** indicates $p < 0.001$.

Table S4 Gender comparison of heat-related mortality risk by heat wave type and period. Data are presented as odds ratios with 95% confidence intervals in brackets. Results show the cumulative effects over lag 0-6 days for different heat wave types (daytime, nighttime, and compound) in 2005-2009 and 2010-2015. All odds ratios represent the comparison between heat wave days and non-heat wave days. *P-value* represents the statistical significance of the difference between male and female results.

**Type**	**Period**	**Male**	**Female**	***P*-value**
Day	2005-2009	0.91 [0.80, 1.02]	0.96 [0.86, 1.08]	0.500
	2010-2015	1.05 [1.02, 1.07]	0.98 [0.96, 1.00]	0.010*
Night	2005-2009	0.96 [0.89, 1.02]	1.03 [0.92, 1.11]	0.300
	2010-2015	1.12 [1.02, 1.30]	1.23 [1.11, 1.43]	0.250
Day-Night	2005-2009	0.92 [0.82, 1.02]	0.95 [0.85, 1.07]	0.700
	2010-2015	1.22 [1.15, 1.36]	1.09 [1.03, 1.13]	0.020*

Table S5 Age comparison of heat-related mortality risk by heat wave type and period. Data are presented as odds ratios with 95% confidence intervals in brackets. Results show the cumulative effects over lag 0-6 days for different heat wave types (daytime, nighttime, and compound) in 2005-2009 and 2010-2015. All odds ratios represent the comparison between heat wave days and non-heat wave days. *P-value* represents whether the oldest age group (>65) has significantly higher odds ratios than all younger age groups combined (marked as <0.05 when significant, blank cells indicate non-significant results).

**Type**	**Period**	**≤ 5**	**5-18**	**18 - 65**	**> 65**	***P*-value**
Day	2005-2010	0.95 [0.89, 1.01]	0.95 [0.89, 1.01]	0.95 [0.87, 1.02]	0.94 [0.83, 1.04]	
	2011-2015	1.09 [1.06, 1.10]	1.04 [0.95, 1.12]	1.09 [1.03, 1.15]	1.00 [0.97, 1.02]	
Night	2005-2010	0.91 [0.80, 1.02]	0.90 [0.74, 1.02]	0.96 [0.92, 1.02]	0.99 [0.87, 1.05]	
	2011-2015	1.13 [1.05, 1.31]	1.10 [1.01, 1.19]	1.14 [1.05, 1.24]	1.27 [1.12, 1.41]	<0.05
Day-Night	2005-2010	0.92 [0.83, 1.01]	0.95 [0.89, 1.02]	1.00 [0.83, 1.18]	0.93 [0.82, 1.02]	
	2011-2015	1.11 [1.04, 1.33]	1.18 [1.06, 1.29]	1.10 [1.02, 1.20]	1.26 [1.13, 1.38]	<0.05

Table S6 Sensitivity Analyses of Heat-Related Mortality Risk by Heatwave Type and Time Period: Model Variations Including $PM_{2.5}$ Adjustment and Alternative Degrees of Freedom (df) for Lag-Response. Data are presented as odds ratios with 95% confidence intervals in brackets. Results show the cumulative effects over lag 0-6 days for different heat wave types (daytime, nighttime, and compound) in 2005-2009 and 2010-2015. All odds ratios represent the comparison between heat wave days and non-heat wave days.

		Daytime	Nighttime	Compound
Unadjusted	2005-2009	0.96 [0.89-1.05]	1.02 [0.87-1.13]	0.94 [0.77-1.04]
	2010-2015	0.96 [0.94-1.01]	1.18 [1.13-1.23]	1.11 [1.03-1.22]
Adjusting for $PM_{2.5}$	2005-2009	0.97 [0.90-1.06]	1.01 [0.86-1.12]	0.94 [0.78-1.05]
	2010-2015	0.95 [0.93-1.00]	1.17 [1.12-1.22]	1.10 [1.04-1.21]
Df for lag-response:				
5	2005-2009	0.95 [0.88-1.04]	1.03 [0.88-1.14]	0.93 [0.78-1.05]
	2010-2015	0.987[0.94-1.02]	1.16 [1.11-1.22]	1.12 [1.05-1.123]

Table S7 Sensitivity analysis of heat wave effects on under-5 mortality using different heat wave definitions. Data are presented as odds ratios with 95% confidence intervals in brackets. Results show the cumulative effects over lag 0-6 days for different heat wave types (daytime, nighttime, and compound) in 2005-2009 and 2010-2015. The unadjusted model uses the baseline definition (90th percentile threshold for 2 consecutive days). Alternative definition is '95% 2d' (95th percentile threshold for 2 consecutive days). All odds ratios represent the comparison between heat wave days and non-heat wave days.

		Daytime	Nighttime	Compound
Unadjusted	2005-2009	0.96 [0.89-1.05]	1.02 [0.87-1.13]	0.94 [0.77-1.04]
	2010-2015	0.96 [0.94-1.01]	1.18 [1.13-1.23]	1.11 [1.03-1.22]
95% 2d	2005-2009	1.10 [0.90-1.18]	0.94 [0.88-1.02]	1.17 [0.94-1.19]
	2010-2015	1.17 [1.10-1.22]	1.35 [1.24-1.53]	1.77 [1.46-2.06]

Table S8 Validation of ERA5-Land Temperature Data (2005–2015) Against Ground-Based Meteorological Stations Near Selected HDSS Sites. Comparison of ERA5-Land daily minimum (T_{min}), maximum (T_{max}), and mean (T_{avg}) temperatures with observed station records during 2005–2015. Validation metrics include root mean square error (RMSE, °C), mean absolute error (MAE, °C), and Pearson correlation coefficients.

Area	Station name	Variable	RMSE (°C)	MAE (°C)	Correlation
Kenya Nairobi	Wilson	T_{min}	1.15	1.12	0.79
		T_{max}	1.07	0.91	0.89
		T_{avg}	0.95	0.74	0.92
Senegal Bandafassi	Kedougou	T_{min}	1.33	1.03	0.87
		T_{max}	1.37	1.19	0.91
		T_{avg}	1.45	1.21	0.89
South Africa Agincourt	Skukuza	T_{min}	1.25	1.21	0.89
		T_{max}	2.42	2.19	0.88
		T_{avg}	1.35	1.03	0.92

Notes: Station observations were obtained from the NOAA Integrated Surface Database (ISD) and the Global Historical Climatology Network (GHCN); Among all HDSS sites, only three locations had meteorological stations within a 50 km radius with complete records for 2005–2015: Nairobi–Wilson (Kenya), Bandafassi–Kedougou (Senegal), and Agincourt–Skukuza (South Africa); ERA5-Land daily data were extracted for the grid cells corresponding to each station location.

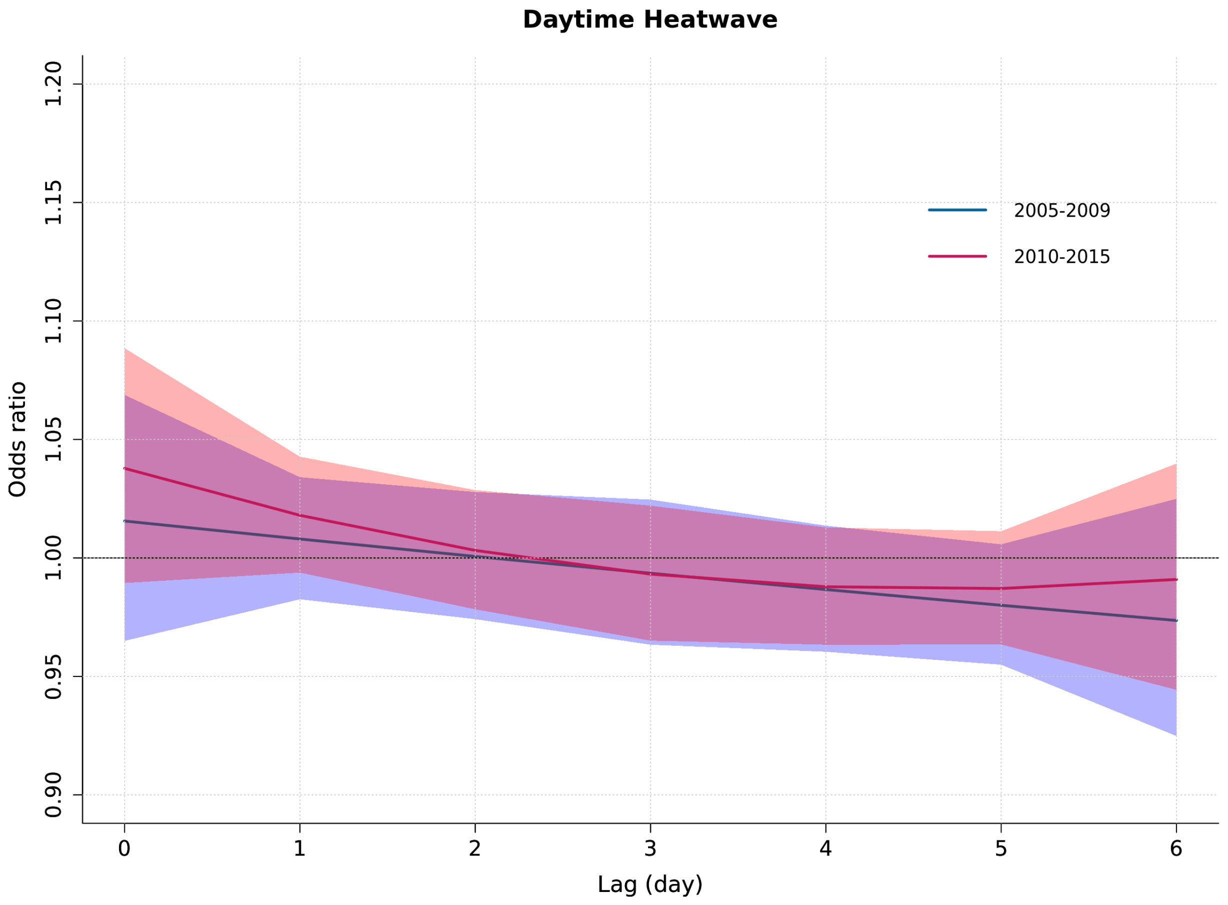

Figure S1 Lag-Response Relationship for Daytime Heatwave Mortality Risk. Comparison of mortality odds ratios following daytime heatwaves (defined as ≥2 consecutive days with maximum daily temperature exceeding the local 90th percentile) between 2005-2009 (blue) and 2010-2015 (red) periods, with 95% confidence intervals shown as shaded areas. The horizontal line at odds ratio = 1 represents no effect.

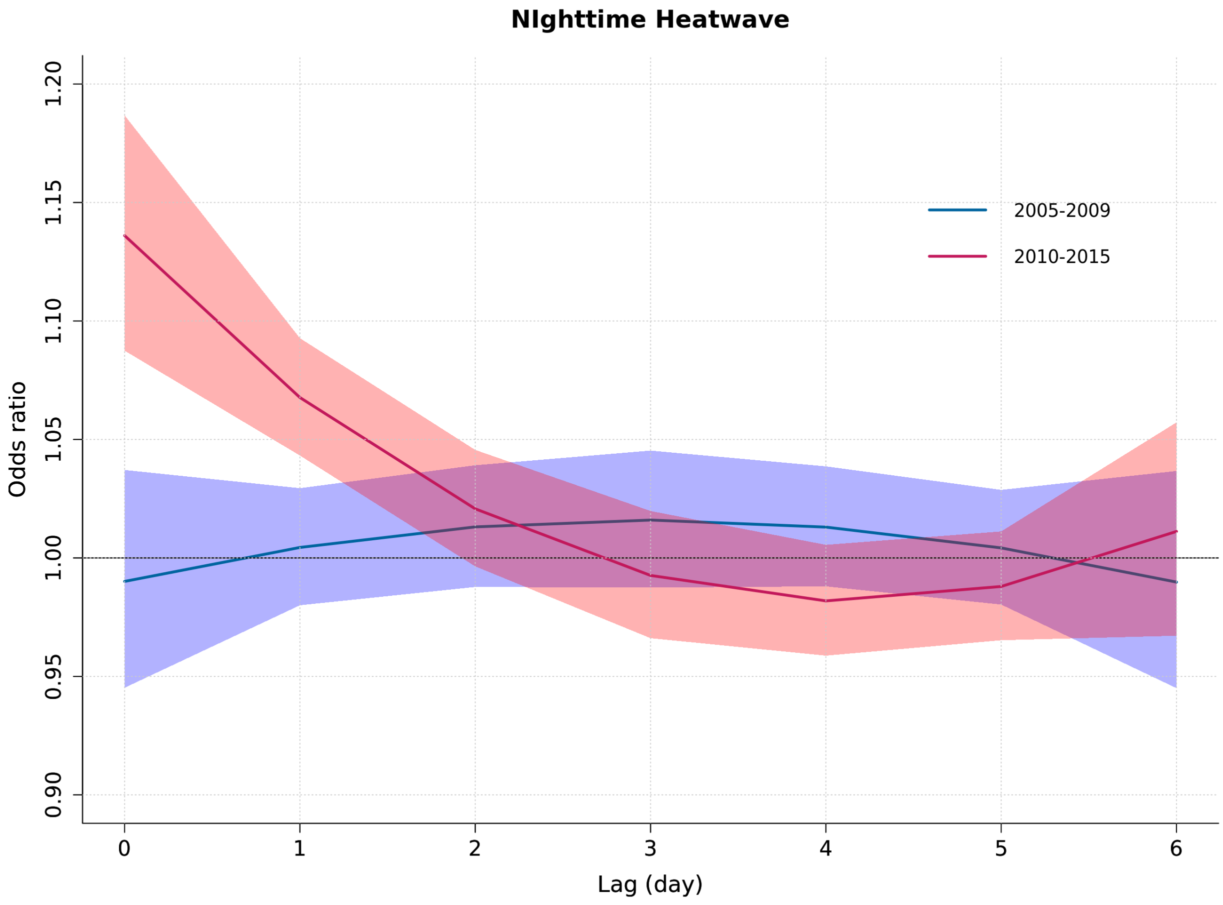

Figure S2 Lag-Response Relationship for Nighttime Heatwave Mortality Risk. Comparison of mortality odds ratios following nighttime heatwaves (defined as ≥2 consecutive days with minimum daily temperature exceeding the local 90th percentile) between 2005-2009 (blue) and 2010-2015 (red) periods, with 95% confidence intervals shown as shaded areas. The horizontal line at odds ratio = 1 represents no effect.

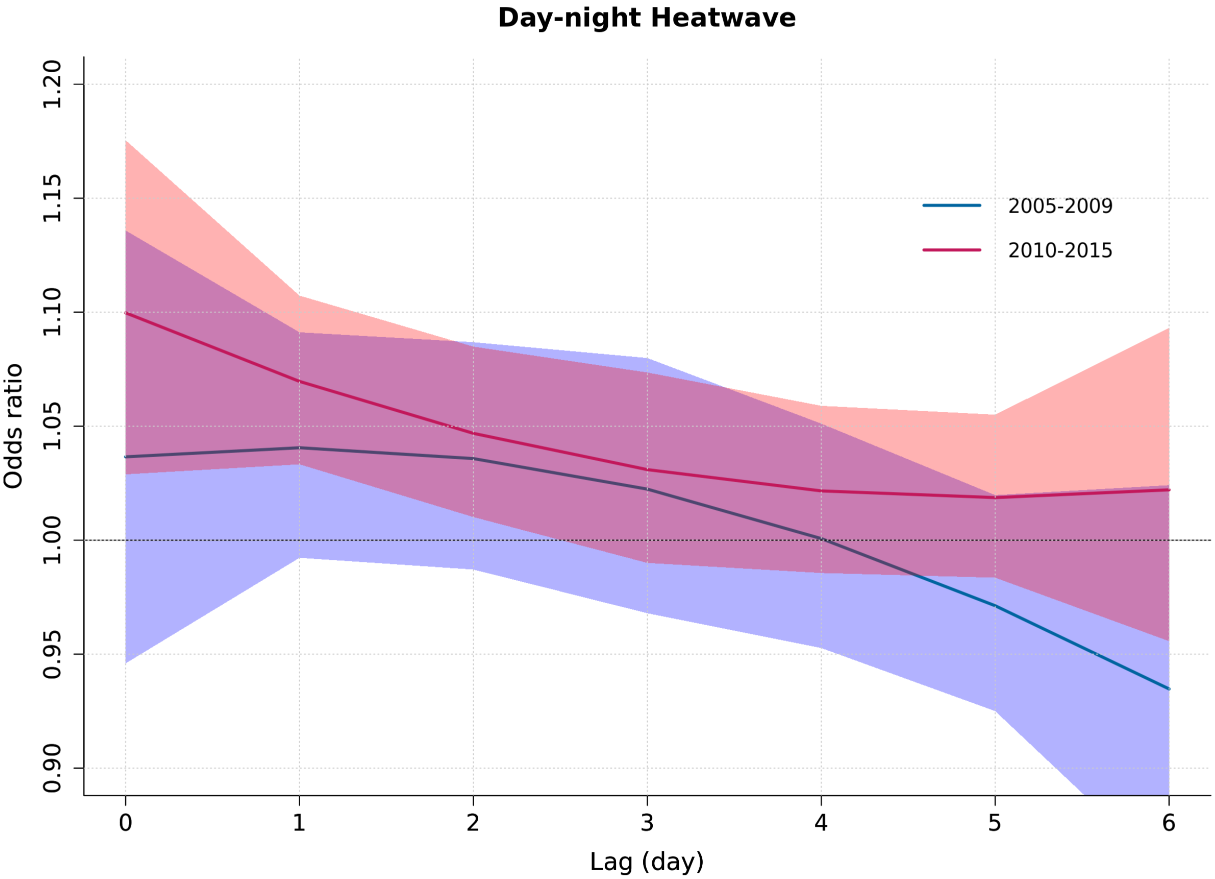

Figure S3 Lag-Response Relationship for Day-Night Compound Heatwave Mortality Risk. Comparison of mortality odds ratios following compound heatwaves (defined as ≥2 consecutive days with both maximum daily temperature and minimum daily temperature exceeding their respective local 90th percentiles) between 2005-2009 (blue) and 2010-2015 (red) periods, with 95% confidence intervals shown as shaded areas. The horizontal line at odds ratio = 1 represents no effect.

REFERENCES AND NOTES

1. R. Vautard, C. Barnes, S. Philip, S. Kew, I. Pinto, F. E. Otto, Heat extremes linearly shift with global warming, with frequency doubling per decade since 1979. *Environ. Res. Lett.* **19**, 094033 (2024).

2. J. S. Kikstra, Z. R. Nicholls, C. J. Smith, J. Lewis, R. D. Lamboll, E. Byers, M. Sandstad, M. Meinshausen, M. J. Gidden, J. Rogelj, E. Kriegler, G. P. Peters, J. S. Fuglestvedt, R. B. Skeie, B. H. Samset, L. Wienpahl, D. P. van Vuuren, K.-I. van der Wijst, A. al Khourdajie, P. M. Forster, A. Reisinger, R. Schaeffer, K. Riahi, The IPCC Sixth Assessment Report WGIII climate assessment of mitigation pathways: From emissions to global temperatures. *Geosci. Model Dev.* **15**, 9075–9109 (2022).

3. C. Lennard, G. Nikulin, A. Dosio, W. Moufouma-Okia, On the need for regional climate information over Africa under varying levels of global warming. *Environ. Res. Lett.* **13**, 060401 (2018).

4. F. Engelbrecht, J. Adegoke, M.-J. Bopape, M. Naidoo, R. Garland, M. Thatcher, J. Mcgregor, J. Katzfey, M. Werner, C. Ichoku, Projections of rapidly rising surface temperatures over Africa under low mitigation. *Environ. Res. Lett.* **10**, 085004 (2015).

5. C. He, S. Breitner, S. Zhang, V. Huber, M. Naumann, C. Traidl-Hoffmann, G. Hammel, A. Peters, M. Ertl, A. Schneider, Nocturnal heat exposure and stroke risk. *Eur. Heart J.* **45**, 2158–2166 (2024).

6. M. G. M. Olde Rikkert, R. J. F. Melis, J. A. H. R. Claassen, Heat waves and dehydration in the elderly. *BMJ* **339**, b2663 (2009).

7. J. Nawaro, L. Gianquintieri, A. Pagliosa, G. M. Sechi, E. G. Caiani, Heatwave definition and impact on cardiovascular health: A systematic review. *Public Health Rev.* **44**, 1606266 (2023).

8. X. Song, S. Wang, T. Li, J. Tian, G. Ding, J. Wang, J. Wang, K. Shang, The impact of heat waves and cold spells on respiratory emergency department visits in Beijing, China. *Sci. Total Environ.* **615**, 1499–1505 (2018).

9. P. Cheveldayoff, F. Chowdhury, N. Shah, C. Burow, M. Figueiredo, N. Nguyen, M. Talbo, R. Jamasi, A. Katz, C. Pasquale, L. Hill, Considerations for occupational heat exposure: A scoping review. *PLOS Climate* **2**, e0000202 (2023).

10. T. Kjellstrom, I. Holmer, B. Lemke, Workplace heat stress, health and productivity–An increasing challenge for low and middle-income countries during climate change. *Glob. Health Action* **2**, 2047 (2009).

11. N. Watts, M. Amann, N. Arnell, S. Ayeb-Karlsson, K. Belesova, H. Berry, T. Bouley, M. Boykoff, P. Byass, W. Cai, D. Campbell-Lendrum, J. Chambers, M. Daly, N. Dasandi, M. Davies, A. Depoux, P. Dominguez-Salas, P. Drummond, K. L. Ebi, P. Ekins, L. F. Montoya, H. Fischer, L. Georgeson, D. Grace, H. Graham, I. Hamilton, S. Hartinger, J. Hess, I. Kelman, G. Kiesewetter, T. Kjellstrom, D. Kniveton, B. Lemke, L. Liang, M. Lott, R. Lowe, M. O. Sewe, J. Martinez-Urtaza, M. Maslin, L. M. Allister, S. J. Mikhaylov, J. Milner, M. Moradi-Lakeh, K. Morrissey, K. Murray, M. Nilsson, T. Neville, T. Oreszczyn, F. Owfi, O. Pearman, D. Pencheon, S. Pye, M. Rabbaniha, E. Robinson, J. Rocklöv, O. Saxer, S. Schütte, J. C. Semenza, J. Shumake-Guillemot, R. Steinbach, M. Tabatabaei, J. Tomei, J. Trinanes, N. Wheeler, P. Wilkinson, P. Gong, H. Montgomery, A. Costello, The 2018 report of the Lancet Countdown on health and climate change: Shaping the health of nations for centuries to come. *Lancet* **392**, 2479–2514 (2018).

12. C. R. Lay, M. C. Sarofim, A. V. Zilberg, D. M. Mills, R. W. Jones, J. Schwartz, P. L. Kinney, City-level vulnerability to temperature-related mortality in the USA and future projections: A geographically clustered meta-regression. *Lancet Planet. Health* **5**, e338–e346 (2021).

13. P. Masselot, M. N. Mistry, S. Rao, V. Huber, A. Monteiro, E. Samoli, M. Stafoggia, F. De’donato, D. Garcia-Leon, J.-C. Ciscar, Estimating future heat-related and cold-related mortality under climate change, demographic and adaptation scenarios in 854 European cities. *Nat. Med.* **31**, 1294–1302 (2025).

14. D. Onozuka, A. Hagihara, Variation in vulnerability to extreme-temperature-related mortality in Japan: A 40-year time-series analysis. *Environ. Res.* **140**, 177–184 (2015).

15. P.-R. Aja, L.-G. Giraudet, S. Houde, in *CONECT. International Scientific Conference of Environmental and Climate Technologies* (2023), pp. 42–42.

16. M. P. Blimpo, M. Cosgrove-Davies, *Electricity Access in Sub-Saharan Africa: Uptake, Reliability, and Complementary Factors for Economic Impact* (World Bank Publications, 2019).

17. E.-H. M. Bah, I. Faye, Z. F. Geh, E.-H. M. Bah, I. Faye, Z. F. Geh, The political economy of housing development in Africa, in *Housing Market Dynamics in Africa*, (2018), pp. 23–55.

18. D. Y. Ayal, Climate change and human heat stress exposure in sub-Saharan Africa, *CABI Reviews* (2021); DOI: https://doi.org/10.1079/PAVSNNR202116049.

19. J. Liu, J. Qi, P. Yin, W. Liu, C. He, Y. Gao, L. Zhou, Y. Zhu, H. Kan, R. Chen, Rising cause-specific mortality risk and burden of compound heatwaves amid climate change. *Nat. Clim. Change* **14**, 1201–1209 (2024).

20. C.-M. Bai, G. Ma, W.-Z. Cai, C.-S. Ma, Independent and combined effects of daytime heat stress and night-time recovery determine thermal performance. *Biol. Open* **8**, bio038141 (2019).

21. T. Weber, A. Haensler, D. Rechid, S. Pfeifer, B. Eggert, D. Jacob, Analyzing regional climate change in Africa in a 1.5, 2, and 3 C global warming world. *Earths Future* **6**, 643–655 (2018).

22. Y. Chung, D. Yang, A. Gasparrini, A. M. Vicedo-Cabrera, C. F. S. Ng, Y. Kim, Y. Honda, M. Hashizume, Changing susceptibility to non-optimum temperatures in Japan, 1972–2012: The role of climate, demographic, and socioeconomic factors. *Environ. Health Perspect.* **126**, 057002 (2018).

23. R. K. Choudhary, P. Joshi, S. Ghosh, D. Ganguly, K. Balakrishnan, N. Singh, R. K. Mall, A. Kumar, S. Dey, Excess mortality risk due to heat stress in different climatic zones of India. *Environ. Sci. Technol.* **58**, 342–351 (2024).

24. K. Minor, A. Bjerre-Nielsen, S. S. Jonasdottir, S. Lehmann, N. Obradovich, Rising temperatures erode human sleep globally. *One Earth* **5**, 534–549 (2022).

25. C. Brimicombe, K. Wieser, T. Monthaler, D. Jackson, J. De Bont, M. F. Chersich, I. M. Otto, Effects of ambient heat exposure on risk of all-cause mortality in children younger than 5 years in Africa: A pooled time-series analysis. *Lancet Planet Health* **8**, e640–e646 (2024).

26. World Meteorological Organization, "State of the Climate in Africa 2023," (World Meteorological Organization, Geneva, 2024).

27. Sustainable Energy For All, Africa's Cooling Challenge Heats Up as Continent Warms. (Sustainable Energy for All, ed. Accessed in 2023 from Sustainable Energy for All website, 2023).

28. J. J. Kunda, S. N. Gosling, G. M. Foody, The effects of extreme heat on human health in tropical Africa. *Int. J. Biometeorol.* **68**, 1015–1033 (2024).

29. C. Wesseling, A. Aragón, M. González, I. Weiss, J. Glaser, C. J. Rivard, C. Roncal-Jiménez, R. Correa-Rotter, R. J. Johnson, Heat stress, hydration and uric acid: A cross-sectional study in workers of three occupations in a hotspot of Mesoamerican nephropathy in Nicaragua. *BMJ Open* **6**, e011034 (2016).

30. International Labour Organization, ILOSTAT Database: Employment by Sex and Economic Activity. (International Labour Organization, ed. Accessed for Nigeria, 2020, under "Employment by Sex and Economic Activity" (ISIC Rev. 4, 2020).

31. Y. Rodgers, H. Akram-Lodhi, *The Gender Gap in Agricultural Productivity in Sub-Saharan Africa: Causes, Costs and Solutions* (UN Women Headquarters Office, 2019).

32. Statistics South Africa, *Quarterly Labour Force Survey* (Statistics South Africa, 2010).

33. E. Igun, X. Xu, Z. Shi, G. Jia, Enhanced nighttime heatwaves over African urban clusters. *Environ. Res. Lett.* **18**, 014001 (2022).

34. M. Itani, N. Ghaddar, K. Ghali, A. Laouadi, Bioheat modeling of elderly and young for prediction of physiological and thermal responses in heat-stressful conditions. *J. Therm. Biol.* **88**, 102533 (2020).

35. W. M. Thiaw, E. Bekele, S. N. Diouf, D. G. Dewitt, O. Ndiaye, M. K. N. Ndiaye, P. N. Ndiaye, N. Diene, M. Diouf, A. Diaw, Toward experimental heat–health early warning in Africa. *Bull. Am. Meteorol. Soc.* **103**, E1843–E1860 (2022).

36. Z. Hausfather, Climate Change Made West Africa's Dangerous Humid Heatwave 10 Times More Likely (2024); https://carbonbrief.org/climate-change-made-west-africas-dangerous-humid-heatwave-10-times-more-likely/.

37. World Meteorological Organization, State of the Climate in Africa 2024 (2025); https://library.wmo.int/records/item/69495-state-of-the-climate-in-africa-2024.

38. P. Murage, B. Anton, F. Chiwanga, R. Picetti, T. Njunge, S. Hassan, S. Whitmee, J. Falconer, H. S. Waddington, R. Green, Impact of tree-based interventions in addressing health and wellbeing outcomes in rural low-income and middle-income settings: A systematic review and meta-analysis. *Lancet Planet. Health* **9**, e157–e168 (2025).

39. M. Rawat, R. Singh, A study on the comparative review of cool roof thermal performance in various regions. *Energy Built Environ.* **3**, 327–347 (2022).

40. K. Herbst, S. Juvekar, T. Bhattacharjee, M. Bangha, N. Patharia, T. Tei, B. Gilbert, O. Sankoh, The INDEPTH data repository: An international resource for longitudinal population and health data from health and demographic surveillance systems. *J. Empir. Res. Hum. Res. Ethics* **10**, 324–333 (2015).

41. O. Sankoh, P. Byass, Cause-specific mortality at INDEPTH Health and Demographic Surveillance System Sites in Africa and Asia: Concluding synthesis. *Glob. Health Action* **7**, 25590 (2014).

42. N. Ng, H. Van Minh, S. Juvekar, A. Razzaque, T. H. Bich, U. Kanungsukkasem, A. Ashraf, S. M. Ahmed, K. Soonthornthada, Using the INDEPTH HDSS to build capacity for chronic

non-communicable disease risk factor surveillance in low and middle-income countries. *Glob. Health Action* **2**, 1984 (2009).

43. P. K. Streatfield, W. A. Khan, A. Bhuiya, S. M. Hanifi, N. Alam, O. Millogo, A. Sie, P. Zabré, C. Rossier, A. B. Soura, HIV/AIDS-related mortality in Africa and Asia: Evidence from INDEPTH health and demographic surveillance system sites. *Glob. Health Action* **7**, 25370 (2014).

44. R. Xu, T. Ye, W. Huang, X. Yue, L. Morawska, M. J. Abramson, G. Chen, P. Yu, Y. Liu, Z. Yang, Global, regional, and national mortality burden attributable to air pollution from landscape fires: A health impact assessment study. *Lancet* **404**, 2447–2459 (2024).

45. J. Muñoz-Sabater, E. Dutra, A. Agustí-Panareda, C. Albergel, G. Arduini, G. Balsamo, S. Boussetta, M. Choulga, S. Harrigan, H. Hersbach, B. Martens, D. G. Miralles, M. Piles, N. J. Rodríguez-Fernández, E. Zsoter, C. Buontempo, J.-N. Thépaut, ERA5-Land: A state-of-the-art global reanalysis dataset for land applications. *Earth Syst. Sci. Data* **13**, 4349–4383 (2021).

46. C. He, H. Kim, M. Hashizume, W. Lee, Y. Honda, S. E. Kim, P. L. Kinney, A. Schneider, Y. Zhang, Y. Zhu, L. Zhou, R. Chen, H. Kan, The effects of night-time warming on mortality burden under future climate change scenarios: A modelling study. *Lancet Planet. Health* **6**, e648–e657 (2022).

47. D. Royé, C. Íñiguez, A. Tobías, Comparison of temperature–mortality associations using observed weather station and reanalysis data in 52 Spanish cities. *Environ. Res.* **183**, 109237 (2020).

48. E. Carracedo-Martínez, M. Taracido, A. Tobias, M. Saez, A. Figueiras, Case-crossover analysis of air pollution health effects: A systematic review of methodology and application. *Environ. Health Perspect.* **118**, 1173–1182 (2010).

49. Y. Zhou, Y. Gao, P. Yin, C. He, W. Liu, H. Kan, M. Zhou, R. Chen, Assessing the burden of suicide death associated with nonoptimum temperature in a changing climate. *JAMA Psychiatry* **80**, 488–497 (2023).

50. C. He, C. Liu, R. Chen, X. Meng, W. Wang, J. Ji, L. Kang, J. Liang, X. Li, Y. Liu, X. Yu, J. Zhu, Y. Wang, H. Kan, Fine particulate matter air pollution and under-5 children mortality in China: A national time-stratified case-crossover study. *Environ. Int.* **159**, 107022 (2022).

51. A. Gasparrini, B. Armstrong, The impact of heat waves on mortality. *Epidemiology* **22**, 68–73 (2011).

52. Y. Guo, A. Gasparrini, B. G. Armstrong, B. Tawatsupa, A. Tobias, E. Lavigne, M. D. S. Z. S. Coelho, X. Pan, H. Kim, M. Hashizume, Y. Honda, Y.-L. L. Guo, C.-F. Wu, A. Zanobetti, J. D. Schwartz, M. L. Bell, M. Scortichini, P. Michelozzi, K. Punnasiri, S. Li, L. Tian, S. D. O. Garcia, X. Seposo, A. Overcenco, A. Zeka, P. Goodman, T. N. Dang, D. Van Dung, F. Mayvaneh, P. H. N. Saldiva, G. Williams, S. Tong, Heat wave and mortality: A multicountry, multicommunity study. *Environ. Health Perspect.* **125**, 087006 (2017).

53. P. Yin, R. Chen, L. Wang, C. Liu, Y. Niu, W. Wang, Y. Jiang, Y. Liu, J. Liu, J. Qi, The added effects of heatwaves on cause-specific mortality: A nationwide analysis in 272 Chinese cities. *Environ. Int.* **121**, 898–905 (2018).

54. A. Gasparrini, Distributed lag linear and non-linear models in R: The package DLNM. *J. Stat. Softw.* **43**, 1–20 (2011).

55. K. Chen, S. Breitner, K. Wolf, R. Hampel, C. Meisinger, M. Heier, W. V. Scheidt, B. Kuch, A. Peters, A. Schneider, Temporal variations in the triggering of myocardial infarction by air temperature in Augsburg, Germany, 1987–2014. *Eur. Heart J.* **40**, 1600–1608 (2019).

56. R. Chen, J. Cai, X. Meng, H. Kim, Y. Honda, Y. L. Guo, E. Samoli, X. Yang, H. Kan, Ozone and daily mortality rate in 21 cities of East Asia: How does season modify the association? *Am. J. Epidemiol.* **180**, 729–736 (2014).

57. K. Bai, K. Li, L. Shao, X. Li, C. Liu, Z. Li, M. Ma, D. Han, Y. Sun, Z. Zheng, LGHAP v2: A global gap-free aerosol optical depth and PM 2.5 concentration dataset since 2000 derived via big Earth data analytics. *Earth Syst. Sci. Data Discuss.* **2024**, 1–29 (2024).

58. A. Gasparrini, Y. Guo, M. Hashizume, E. Lavigne, A. Zanobetti, J. Schwartz, A. Tobias, S. Tong, J. Rocklöv, B. Forsberg, M. Leone, M. de Sario, M. L. Bell, Y.-L. L. Guo, C.-f. Wu, H. Kan, S.-M. Yi, M. d. S. Z. S. Coelho, P. H. N. Saldiva, Y. Honda, H. Kim, B. Armstrong, Mortality risk attributable to high and low ambient temperature: A multicountry observational study. *Lancet* **386**, 369–375 (2015).
